# The Foreign Journals: Surgery

**Published:** 1842-10

**Authors:** 


*Gazette des Hôpitaux. Avril* 16, 1842.

### SURGERY.

### *On the Mechanism of Spontaneous or Symptomatic Luxations of the Femur.* By M. J. Parise.

The author has in this paper very clearly and fully illustrated the opinion that collections of fluid, serous, synovial, or purulent, in the hip-joint are the ordinary cause of the symptomatic dislocation of the femur. We believe he takes too exclusive a view of the matter, and too much disregards other causes of luxation, as well as those cases in which, though the femur is still in the acetabulum, there are yet the signs of luxation; but of this class of luxations by accumulations of fluid, we have not before read so good an account. We shall therefore extract the newest and most important parts of it.

He overthrows satisfactorily all the objections advanced against E. H. Weber's account of the influence of the atmospheric pressure in maintaining the head of the femur in its place, and then shows the influence of artificial injections of the cavity of the hip-joint in forcing out the head of the femur. His plan was to bore through the thinnest part of the acetabulum obliquely, and then to inject fluid into it; the quantity which the capsule would hold being from forty to forty-five grammes of water. The constant results were: 1st, the femur was carried in the direction of flexion, abduction, and rotation outwards; the flexion being equal to an angle of from 30° to 35° with the plane of the horizon, and the rotation outwards being very slight; 2d, the great trochanter was forced outwards, and separated from the anterior superior spine of the ilium, and from the symphisis pubis, for more than half an inch; and was, at the same time, forced downwards, so that the limb was elongated about half an inch; 3d, the capsule was not distended equally, but was especially enlarged near the trochanters; and lastly the head of the femur was driven so far out of the acetabulum, that the distance of its summit from the bottom of the cavity, (as measured on an injection which solidified on cooling,) was upwards of two-thirds of an inch. The round ligament seemed to have but little influence on this displacement.

He relates an interesting case of a child twelve years old, in which it is quite clear that the luxation of the femur, where most of the signs of ordinary disease of the hip-joint had been present, was due to the accumulation of a serous fluid mixed with albuminous flocculi within the hip-joint. The cotyloid cavity and the head of the femur were uninjured, except that the latter was grooved by the pressure which it had experienced against the edge of the acetabulum. He believes that at a later period there would have been in this case the same disease of the bones and cartilages as is commonly seen in dislocations after disease of the hip-joint: [but this is, we think, very improbable; the femur and acetabulum would most probably have undergone only the same changes as after dislocation by external force. There is no proof here that the ordinary changes in diseased hips are, as the author suggests, consecutive to the dislocation.]

He enters upon a very elaborate yet clear account of the mode in which a fluid slowly secreted into the capsule of the hip-joint must act, and how it must produce, (as is evident from his experiments already mentioned,) first, elongation of the limb, real though but slight, and then the condition in which by the action of the muscles the femur may be drawn upwards and backwards. The peculiar positions of the limb, and the succession of their changes, he refers to three chief conditions: 1st. The dilatation of the capsule, which being inversely proportionate to the powers of resistance of its several parts, must be least in the situation of the accessory ligament on its anterior and inner aspect. This will restrain the movement of the head of the femur, which will therefore, as the capsule is distended, be carried outwards and upwards, while the shaft moves inwards and is flexed. 2d. The action of the pelvi-femoral muscles, of which those that lie next the capsule are like it distended by the accumulating fluid, and when they contract, since they all contract with nearly equal force, can have but little effect; while those which lie at a greater distance from the capsule, the adductors, glutei, &c. must by their contraction exercise an important influence in producing both the flexion and adduction, and, at last, the dislocation upwards and backwards of the limb. 3d. The position which the patient naturally assumes, and which is that best adapted for the avoidance of pressure on the diseased joint. For this purpose, he inclines over to the healthy side, and flexes his thigh and turns it inwards, in order that it may rest more easily on the other. At first, also, the round ligament assists in producing the flexion and adduction; and it resists, at the same time, the passage of the head of the femur upwards and backwards, but at length it gives way, and if the femur is pushed sufficiently outwards from the acetabulum, there is scarcely anything to prevent the muscles from pulling it upwards.

Lastly, the author explains the changes which he supposes to take place in the head of the femur after its luxation; [but these we think (as we have already said) he overrates, by presuming that a mere collection of fluid in the hip-joint without disease of the bones or cartilages is a common occurrence.]

*Archives Générales de Médecine. Mai et Juin,* 1842.

---

*On a new Cranial Saw.* By M. BERTHERAND.

THE author has invented a saw for cutting the cranium, in examinations after death, and calls it a *cranial cyclotome.* It consists of a saw, concave at one edge and convex at the other, which, by means of a screw, can be turned in its long handle, so as to present either edge at will in the direction for cutting. It is also fitted with a copper conductor, which can be worked in the same way, and fixed at any required distance from the edge, so as to prevent the saw from passing too deeply through the skull.

*Gazette des Hôpitaux. Juin* 23, 1824.

---

*Passage of Air into the Veins.* By DR. ASMUS.

THE author was removing a "steatoma" as large as the two fists from the region between the lower jaw and clavicle of a man forty years old, and was very carefully separating its base from the carotid artery with which it was in contact, when he accidentally opened the internal jugular vein, which had been pushed far from its usual place by a lobe of the tumour. No blood flowed; but on the instant he heard air enter the vein with a bubbling sound. He asked the man how he felt, who said "Well;" but in the next moment cried out, "Its all up!" and began to be convulsed, first in the face, and then in the whole body. He sunk down, and at the same instant another bubble was heard; but still no blood flowed. Alternate convulsive movements and opisthotonos ensued; the face was deadly pale, the breath short, and death seemed close at hand. Rapid bleeding now took place from the wound, and a stream of black blood was seen to issue from the vein, but as often as the patient was convulsed, air again passed in, and the bubbling was distinctly both seen and heard. A ligature was as quickly as possible put upon the vein above the injured part, and with this the bubbling ceased; the tumour was cut off level and the patient was put to bed.

Syncope, alternating with severe convulsions, still continued; the pulse was not discernible, the heart seemed only to vibrate, and the respiration was short. Stimulants and a variety of restorative means were employed, and about twelve hours after the operation (in which the loss of blood was altogether moderate), the patient began to revive. His condition continued to improve, and he at length completely recovered. [This appears a well-marked case. At the time of opening the vein the patient had lost less than five ounces of blood, and was cheerful; at the instant the bubbling was heard the symptoms began, and the passage of air into the vein was distinctly observed by the eye and ear.]

*Medicinische Zeitung. Juni* 8, 1842.

---

*Intra-parietal Hernia after a Wouud of the Abdomen.* By M. Berard.

The case detailed in M. Berard's clinical lecture affords a good example of an accident which is apt to occur not only in penetrating wounds of the abdomen, but in operations for hernia. In the endeavour to force the intestine (which had protruded) back into the abdomen, it was pushed up between the layers of abdominal muscles, and here, in the cavity thus artificially formed, became strangulated. The case was the more perplexing, because, when the intestine was in this position, the finger could be easily passed into the abdomen, and the intestine seemed to be entirely reduced.

*Gazette des Hôpitaux. Juin* 28, 1842.

---

*Case of Strangulated Hernia through the foramen thyroideum.*
By Dr. Frantz, of Genthin.

The patient was a strong woman, forty years old, whom the author found with many of the signs of strangulated hernia, and complaining of a severe pain at the upper and inner part of the left thigh, which had come on suddenly and was increased in paroxysms at intervals of about ten minutes. There was no redness, heat, or swelling at the part, but on pressing the point of the finger high up between the triceps and adductor muscles, severe pain was produced. There was pain, but no tenderness, of the abdomen. The patient had long had double femoral hernia, but neither of these was now down. Three years before, she had had signs exactly like the present, but had been suddenly relieved when, as she was pressing upon the part, something seemed to go back with a noise into the abdomen. Since that time the same symptoms had occasionally recurred in a less degree, but they had been always relieved by the same plan of pressing, as if to reduce a hernia. On the present occasion, however, they were much more severe; bleeding, purgatives, repeated applications of pressure, and various other remedies were tried in vain. On the fourteenth day the signs of strangulation having regularly increased, and stercoraceous vomiting having existed since the ninth, the patient seemed to be quickly dying, when, to the surprise of all, a spontaneous evacuation of fæces took place, and she began slowly to recover. Her recovery was ultimately complete.

*Allgemeine Medicinische Central-Zeitung. April* 27, 1842.

---

*Remarkable Case of Thoracic Fistula.* By Professor Retzius, of Stockholm.

The patient is a clarionet-player, who notwithstanding this diseased state, can follow his avocation and take long walks. He closes the opening in his side with a cork; and as often as he *uncorks* himself, air mixed with pus passes freely in and out through the aperture.

*Tidskrift för Läkare,* 1839, and *Schmidt's Jahrbucher,* No. 3, 1842.

*Surgical Use of the Magnet.*

In the workshops of Fairbairne (in Belgium) there has been recently put up an artificial magnet of great power, at the level of the eye. Every instant one may see a turner, or an adjuster, or some other kind of workman, who has had a particle of iron driven into his eye, running to the magnet, which draws it out as soon as the eyelids are separated and the eye is held near its pole. One may conceive from this how a magnet might be made of sufficient power to draw a piece of iron even from the flesh or from the bones.

*Gazette des Hopitaux. Juin* 14, 1842.

---

*Preservation of Nitrate of Silver.* By M. DUMERIL.

For this purpose M. Dumeril has invented a plan. He melts in a vessel over a fire the best sealing-wax, containing a large quantity of gum-lac, and in this he dips the piece of caustic, over which he thus obtains a complete and firmly adherent varnish impermeable by the air or light, and completely preventing the staining of the fingers. In using the caustic all that is necessary is to scrape off with a penknife as much of the wax as is required to expose a surface of given size. Caustic thus coated is of course very convenient for introducing into cavities of which only a small portion is to be touched.

*Bulletin Gén. de Thérapeutique. Mai,* 1842.

---

*A case of Exophthalmia, with Œdema of the Conjunctiva, and Opacity of the Crystalline Lens in a Puerperal Woman.* By M. BLANDIN.

A woman, forty-one years old, was delivered, after a tedious labour, on December 3d, 1841. For fifteen days no unusual symptom occurred, but on the sixteenth and seventeenth day the patient was attacked by a violent shivering fit. On the eighteenth day, however, she returned from the hospital to her own home. and for some days afterwards suffered from febrile attacks, though they were no longer preceded by severe shivering. From the 25th of December the right eye began to project, the patient suffering little beyond a sense of weight in the head, principally in the supra orbitar region. Vision was at first unimpaired, but failed as the exophthalmia increased, and at last the patient became quite blind of that side. In this condition the patient applied to M. Blandin, at the Hôtel Dieu. There was then considerable prominence of the right eye, the conjunctiva of the globe was prominent, red, and swollen, and evidently infiltrated. The cornea was natural, the aqueous humour retained its transparency, and there was no evident change in the structure of the iris, but it had lost its contractility, and the eye was uninfluenced by exposure to a strong light. The crystalline lens appeared opaque, and of a shining, milk-white colour; the anterior membranes of the lens being in all probability the seat of the opacity. The volume of the globe was normal, the pain in the affected parts was inconsiderable, and no tumefaction existed of the parotid or cervical glands. The intellectual faculties were perfect and the general health was good.

In his remarks on the case M. Blandin offers some observations on the diagnosis of the affection. Some ramifications of the conjunctiva gave exit to a small quantity of pus from its inferior external portion. From that time the eye gradually retreated into the orbit, and from these circumstances M. Blandin concludes that there existed a small abscess behind the eye. The cause of the formation of this abscess is open to debate. It might be one of those purulent deposits occasionally met with in puerperal women. M. Blandin, however, regards it rather as the result of phlebitis, probably of the ophthalmic vein. He is likewise disposed to regard the affection as altogether analagous to *phlegmasia dolens,* in which disease the femoral vein becomes obliterated, just as here, in all probability, the ophthalmic vein was. On any other supposition the opacity of the capsule of the crystalline lens does not admit of explanation; while, in two other instances in which this lesion of the ophthalmic vein was discovered after death, precisely this condition of the crystalline lens had been noticed during the lifetime of the patient. *Gazette des Hôpitaux. Jan.* 27, 1842.

*Observations on Fibrous Polypi of the Uterus.* By M. A. Berard, of the Hôpital Necker.

On the occasion of a patient labouring under polypus of the uterus being admitted into the hospital, M. Bérard made some observations on the management of these growths.—The patient was a single woman, 47 years old, who had good health and menstruated regularly from the age of 11 to 24. From that time, however, till her 44th year menstruation became very scanty, but afterwards returned, being exceedingly abundant, and at length flowed continually in greater or less quantity. From this constant loss of blood the patient beeame at length much exhausted, and was admitted into the hospital in a state of complete anæmia. An examination per vaginam discovered the neck of the uterus to be distended, its orifice directed to the left side and slightly open, and the lips of the os uteri were thinned, and its cavity was occupied by a rounded oblong tumour, which was ascertained to be attached to the interior of the uterus.

With reference to the symptoms that were observed, M. Bérard remarks that Levret is mistaken in supposing that hemorrhage occurs only when the tumour having cleared the cervex uteri and descended into the vagina, the circulation in it becomes impeded by reason of the constriction of the polypus by the neck of the uterus. He is on the other hand disposed to regard these hemorrhages as active, and analogous to those which take place during the course of abortion, an analogy the more strongly marked, since in the one case as in the other uterine contractions take place. From a consideration of the symptoms he proceeds to an inquiry into the proper treatment, and decides in this case in favour of excision rather than of the ligature. The dangers of excision he regards as trivial while the great superiority which it possesses over the ligature in the speedy removal of the growth is universally admitted.

The operation which proved completely successful, was performed by placing the patient in the position for lithotomy. Two assistants kept the thighs properly bent, while a third made pressure on the hypogastric region. The operator having introduced a bivalve speculum, incised the two commissures of the cervix uteri with a probe-pointed bistoury, which permitted the polypus to enter the vagina. The speculum was then withdrawn, and two hooks being placed in the tumour it was gradually drawn down to the vulva. The operation was then completed by excising the pedicle of the polypus with a pair of curved scissors. The patient perfectly recovered.

*Gazette des Hopitaux. Jan.* 8, 1842.

---

*On the Treatment of Leucoma by Incisions into the Cornea.* By Dr. Holscher, of Hanover.

Two cases are related in which this treatment was adopted. The case which suggested it was that of a girl, twenty-two years old, who had lost the left eye from purulent ophthalmia in infancy, and in whom the right was almost blind from leucoma of nearly the whole cornea. Various means had been used in vain. The author, therefore, made an artificial pupil by drawing the iris through the cornea and excising a portion of it. Severe inflammation ensued which was with difficulty managed; but three months after, the patient not only had a good artificial pupil, but the cornea had become much less leucomatous, and this especially at the part where the incision through it had been made. The next bad case of leucoma, therefore, which the author met with, he treated as follows: The patient was a lad fourteen years old, who had lost his right eye from purulent ophthalmia in infancy, and had leucoma of nearly all the left cornea. At four different times, with intervals of eight days, a common cataract knife was passed into the cornea as deep as possible without penetrating it, and was drawn out with a sliding motion. After the first three times no inflammation ensued; therefore, after the fourth, some tinct. opii was dropped into the wound three times a day. Severe inflammation set in, but it was moderated by local bleeding, and the treatment by opium was continued for two months. The leucoma became gradually less, and the patient who could at first only discern light from darkness, became able to guide himself in walking, and to perceive the window-frames in his room. The second case was that of a man forty years old, who had leucoma of one eye from gonorrhœal ophthalmia. It had been variously but vainly treated for a year. The author made incisions into the cornea twice, with an interval of fourteen days. After the second, a tolerably severe inflammation ensued which required active treatment. As soon as it had ceased, sulphate of zinc and tincture of opium were again dropped into the eye, and after a year and a half, not a trace of leucoma could be seen.

*Holscher's Hannoversche Annalen. September,* 1841.

---

*On the local Employment of Calomel in Ophthalmia Neonatorum.* By M. Lauer.

The introduction of finely-powdered calomel into the eye in ophthalmia was originally practised by Dupuytren. Professor Fricke of Hamburg has noticed in his Zeitschrift for 1837, the violent reaction which its employment excites in the case of persons who have been taking iodine, probably owing to the formation of an iodide of mercury. About a year since Dr. Kluge began to use it as a local application in the cases of ophthalmia of new-born infants which came under his care in the lying-in department of the Charité at Berlin. The results were extremely fortunate, and Pr. v. Siebold of Göttingen, who was induced to try the remedy, has obtained from its employment very great success.

The manner of introducing the calomel into the eye is by means of a camel's hair pencil loaded with the powder, which is shaken from it into the eye, while an assistant separates the lids. In the treatment of the ophthalmia neonatorum this remedy may be had recourse to as soon as the first traces of the disease appear, and its employment once daily is then in general sufficient. After the lapse of from half an hour to two hours, according to the quantity of the secretion, the eye may be washed from the powder, and the ordinary rules as to cleanliness be attended to. In severe cases the application may be repeated twice every day; but when the disease is mild a single application daily suffices to effect a cure in from four to ten days, if the remedy had been had recourse to from the outset. The more severe and intractable forms of the disease do not appear to have been benefited by the local employment of calomel.

*Medicinische Zeitung. Juni* 8, 1842.

